# High-Gain Miniaturized Ultrawideband Antipodal Vivaldi Antenna with Metamaterials

**DOI:** 10.3390/mi17010008

**Published:** 2025-12-21

**Authors:** Wentao Zhang, Linqi Shi, Chenjie Zhao, Rui Yang

**Affiliations:** 1 China Airborne Missile Academy, Luoyang 471009, China; 2 National Key Laboratory of Air-Based Information Perception and Fusion, Luoyang 471009, China; 3 National Key Laboratory of Radar Detection and Sensing, School of Electronic Engineering, Xidian University, Xi’an 710071, China; 24241215297@stu.xidian.edu.cn (L.S.); m17362028614@163.com (C.Z.)

**Keywords:** antipodal Vivaldi antenna, ultrawideband (UWB) antenna, metamaterial, high-gain, miniaturization

## Abstract

A compact high-gain antipodal Vivaldi antenna with ultra-wideband (UWB) performance ranging from 1 GHz to 25 GHz is proposed and demonstrated. The antenna features two sets of tapered exponential slots along the flare edges to enhance low-frequency impedance matching and broaden the operating bandwidth. To address high-frequency gain degradation, a rhombus-shaped metamaterial array is embedded within the tapered slot region, effectively improving radiation directivity and suppressing gain roll-off without enlarging the antenna footprint. Full-wave simulations and experimental measurements confirm that the proposed antenna achieves a well-matched impedance bandwidth from 1 to 25 GHz, with a peak gain of 15.84 dBi. Notably, the gain remains consistently above 14 dBi in the high-frequency region, verifying the effectiveness of the embedded metamaterial structure. The proposed design successfully balances wideband operation, high gain, and compact form factor, offering a promising solution for space-constrained UWB applications in communication, sensing, and imaging systems.

## 1. Introduction

Ultra-wideband (UWB) antennas have found widespread applications in various domains, including wireless communications [1,2,3], radar detection [4,5], and geological exploration [6], owing to their distinct advantages such as large channel capacity, low power consumption, and high data transmission rates. Among various UWB antenna configurations, the Vivaldi antenna [7,8] has garnered significant attention due to its simple structure, high directivity, and inherent broadband characteristics.

In practical systems, Vivaldi antennas and their antipodal variants have been widely employed in UWB radar and imaging, ground-penetrating and through-wall sensing, spectrum monitoring, and emerging high-frequency short-range links. In these scenarios, the antenna is required to provide not only a very wide impedance bandwidth but also sufficiently high and stable gain, controlled sidelobe levels, and a compact planar profile that can be easily integrated into arrays or front-end modules. These application-driven requirements make it particularly challenging to simultaneously maintain ultrawideband performance, high gain, and miniaturized size in a single Vivaldi design.

Numerous Vivaldi antenna designs have been proposed to enhance operational bandwidth, improve radiation efficiency, and reduce physical dimensions. For instance, impedance loading techniques can be used to extend the bandwidth [9,10], while geometric modifications—such as fern-fractal flares [11], tapered-slot edge loading [12,13,14] and serrated contours [15,16]—have been explored to suppress the low-frequency cutoff and improve impedance matching. More critically, there exists a fundamental trade-off in Vivaldi antenna design: the simultaneous realization of miniaturization and ultra-wideband operation remains highly challenging. Structural modifications aimed at reducing antenna size typically compromise gain or bandwidth, limiting their effectiveness in compact or integrated systems. Conversely, designs that ensure high gain across the broad operating bandwidth often involve bulky configurations, thereby sacrificing system compactness and integration potential.

On the other hand, achieving wideband performance often comes at the cost of degraded gain at higher frequencies where the radiation efficiency tends to drop significantly. To address high-frequency gain degradation, researchers have introduced parasitic elements in the flaring region, such as metallic directors [17] and metamaterial-based lenses [18,19], to focus energy and improve directivity. While these strategies help enhance performance in specific frequency ranges, they usually emphasize either bandwidth or gain, rarely achieving both in a compact and efficient configuration. Even when designs do meet both criteria [20], the increase in overall dimensions diminishes their practical utility for space-constrained applications.

Motivated by these considerations and the stringent requirements of practical UWB radar, sensing, and communication platforms, this work proposes a miniaturized, high-gain antipodal Vivaldi antenna with ultrawideband performance from 1 to 25 GHz. Two sets of exponentially tapered edge slots are introduced along the flare region to enhance impedance matching, extend the operational bandwidth, and simultaneously reduce the antenna size. Furthermore, to address the challenge of gain instability and pattern distortion at the upper end of the UWB spectrum, a rhombus-shaped metamaterial excitation structure is embedded within the tapered slot region. This structure effectively assists wavefront shaping, enhances radiation directivity, and suppresses high-frequency gain roll-off without increasing the antenna footprint. As a result, the proposed antenna achieves a balanced combination of bandwidth, gain, and compactness that is attractive for real-world UWB systems.

## 2. Antenna Design and Numerical Results

**Figure 1:**
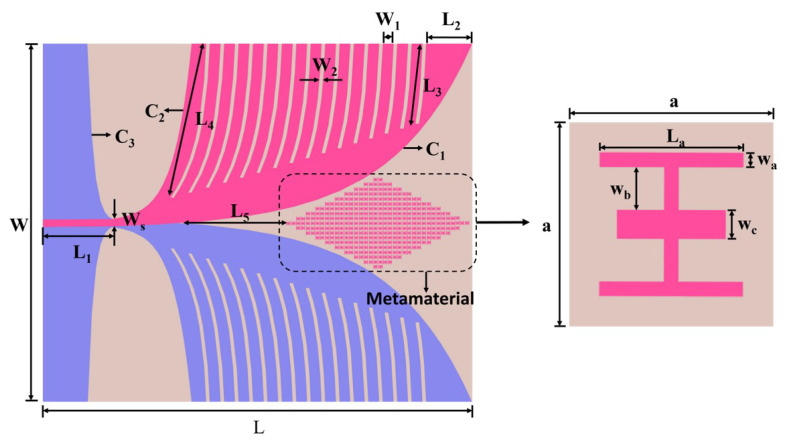
Configuration of the proposed ultrawideband antipodal Vivaldi antenna. (**a**) Overall geometry of the antenna, showing the rhombus-shaped metamaterial array embedded in the tapered-slot region. The dashed outline indicates the loading area of the metamaterial array. (**b**) Metamaterial unit-cell.

The detailed geometry and overall dimensions of the proposed antenna are shown in Figure 1. The dimensions of all antennas are 150 mm × 180 mm (0.5λ×0.6λ), where λ is the wavelength at the lowest cutoff frequency. The conductors are printed on both sides of the F4B dielectric substrate ($\varepsilon_{r}=2.65$, tanδ=0.002). The flared opening of the radiator follows an exponential curve, which is defined by the following expression:

(1) $$\begin{array}{c} C_{1}:x\left(t\right)=e^{c_{1}t}-1-w_{s}/2,y\left(t\right)=t+l_{1} \end{array}$$

(2) $$\begin{array}{c} C_{2}:x\left(t\right)=e^{c_{2}t}+1-w_{s}/2,y\left(t\right)=t+l_{1} \end{array}$$

(3) $$\begin{array}{c} C_{3}:x\left(t\right)=e^{c_{3}t}-1-w_{s}/2,y\left(t\right)=-t+l_{1} \end{array}$$

where $w_{s}$ = 2.85 mm, $L_{1}$ = 30 mm, $c_{1}$ = 0.0289, $c_{2}$ = 0.13, and $c_{3}$ = 0.4. Other parameters are summarized in Table 1.

The proposed antenna incorporates two sets of tapered exponential slots along the edges of the exponential aperture to enhance impedance matching and extend the low-frequency bandwidth. In addition, a metamaterial array is embedded within the aperture region to improve high-frequency gain and radiation directivity. These structural features contribute to achieving a wide impedance bandwidth, high gain performance, and compact size across the 1–25 GHz operating range.

**Figure 2:**
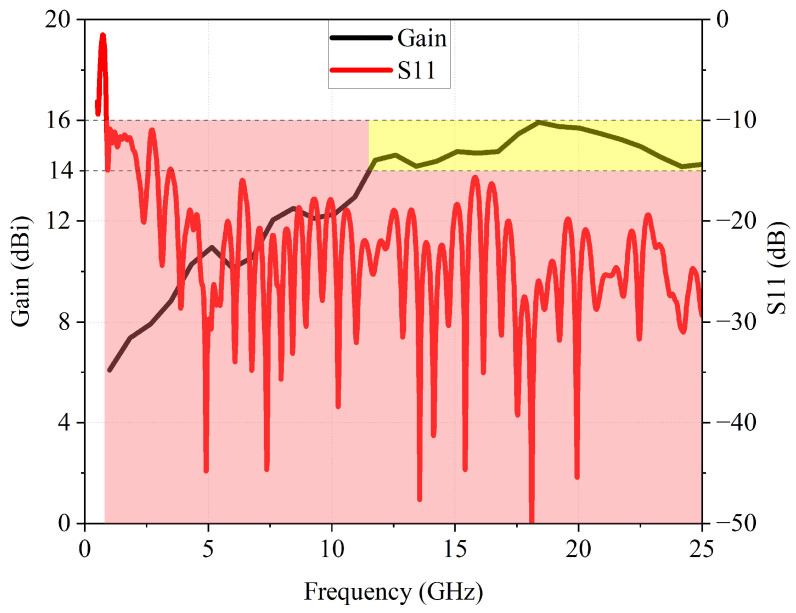
Simulated $S_{11}$ and gains of antenna.

**Figure 3:**
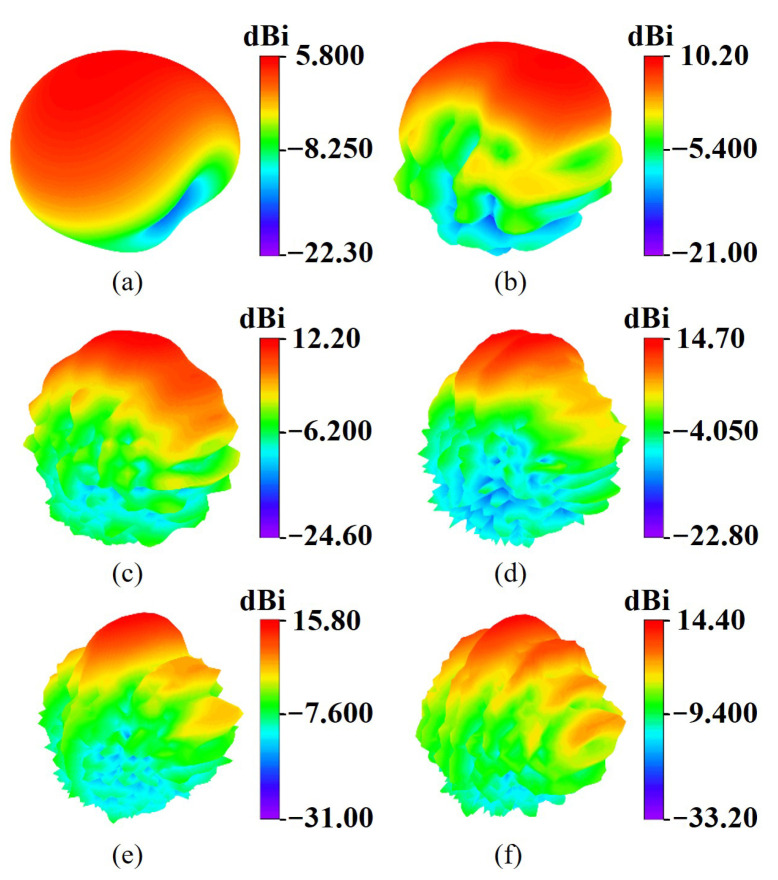
3D radiation patterns of antenna at (**a**) 1, (**b**) 5, (**c**) 10, (**d**) 15, (**e**) 20, (**f**) 25 GHz.

We carry out the full wave simulations (CST Studio Suite) to verify the proposed design, as shown in Figure 2 and Figure 3. As observed in Figure 2, the antenna achieves an ultra-wide operating bandwidth ranging from 1 to 25 GHz, while maintaining a gain above 14 dBi from the mid-frequency range of 11.5 GHz. Figure 3 illustrates the antenna 3D radiation pattern, which remains stable with well-defined main lobes across the band. Notably, the antenna effectively suppresses beam splitting at high frequencies, ensuring enhanced directivity and consistent radiation performance.

**Figure 4:**
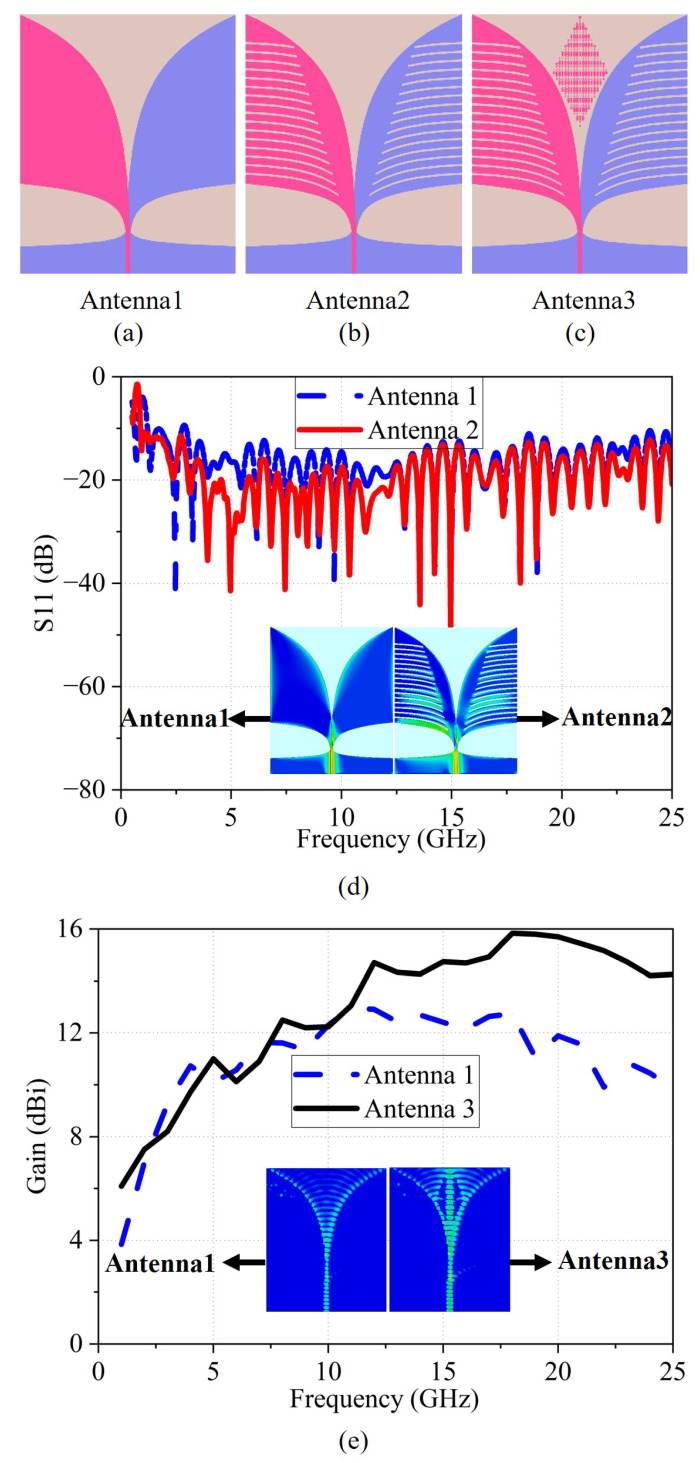
(**a**) Traditional antipodal Vivaldi design, (**b**) Vivaldi antenna with tapered slots, (**c**) the proposed antipodal Vivaldi antenna, and (**d**) simulated $S_{11}$ of different antennas; the inset shows the surface current distributions of Antenna 1 and Antenna 2 at 1 GHz, (**e**) simulated gain of different antennas; the inset shows the electric-field distributions of Antenna 2 and Antenna 3 at 20 GHz.

The evolution of the antenna, as illustrated in Figure 4a–c, begins with an antipodal Vivaldi design featuring a 50 Ω microstrip feed, which shows limited low-frequency response and suffers gain degradation at higher frequencies. To broaden the operational bandwidth and improve impedance matching, two sets of tapered exponential slots are introduced along the edges of the exponential aperture. These slots lengthen the current path and significantly enhance low-frequency performance. Next, to boost high-frequency gain and refine radiation directivity without altering the antenna’s compact form, a metamaterial array is embedded within the aperture region. Electromagnetically, the metamaterial loading can be interpreted as an inhomogeneous effective medium placed in the tapered-slot region. The periodic metallic inclusions modify the local effective permittivity and phase velocity, so that the guided wave is slightly slowed down within the loading area. This produces an additional phase delay that compensates for the aperture truncation and helps equalize the phase front before radiation. As a result, the wavefront at the aperture becomes better collimated towards the endfire direction, which directly contributes to the enhanced high-frequency gain and improved radiation directivity without requiring a larger physical aperture.

Figure 4d,e present the simulated $S_{11}$ and gain responses over the 1–25 GHz frequency range. As shown, Traditional antipodal Vivaldi design exhibits an $S_{11}$ of –4 dB at 1 GHz and values exceeding –10 dB between 2.77 and 2.96 GHz, indicating a lower operational frequency limit of approximately 3 GHz. Additionally, its gain decreases significantly beyond 18 GHz. Vivaldi antenna with tapered slots achieves an $S_{11}$ below –10 dB across the entire 1–25 GHz range, effectively extending the lower cutoff frequency to 0.88 GHz. However, the gain performance remains comparable to that of the previous configuration, showing a similar degradation at higher frequencies. The proposed Antipodal Vivaldi Antenna demonstrates substantial gain enhancement, with the gain increasing significantly from 2 GHz onward and maintaining levels above 14 dBi throughout the 12–25 GHz range. A maximum gain improvement of 4.96 dBi is achieved relative to the previous stage of the antenna design.

To clearly demonstrate the improvement in low-frequency performance, Figure 4d presents the surface current distributions of Antenna 1 and Antenna 2 at 1 GHz. The surface current in Antenna 1 is primarily concentrated along the edges of the radiator. With the introduction of tapered exponential slots, the current distribution in Antenna 2 is modified, showing evident current flow through the slot regions. This extension of the current path contributes to enhanced radiation at low frequencies.

To further elucidate the mechanism behind the high-frequency gain enhancement, Figure 4e illustrates the electric-field distributions of Antenna 2 and Antenna 3 at 20 GHz. For Antenna 2, the electric field near the tapered-slot region is relatively weak and exhibits a less uniform distribution across the aperture, indicating that part of the guided energy is not efficiently converted into endfire radiation. In contrast, the integration of the metamaterial structure in Antenna 3 alters the wavefront characteristics, strengthens the field confinement in the tapered-slot region, and produces a more uniform field distribution along the aperture. This behaviour suggests that the metamaterial-loaded section acts as an effective inhomogeneous medium that guides and reshapes the wavefront toward the endfire direction, thereby reducing diffraction and leakage along the edges. Consequently, more power is steered into the main beam, which explains the notable gain enhancement observed for Antenna 3 at high frequencies.

**Figure 5:**
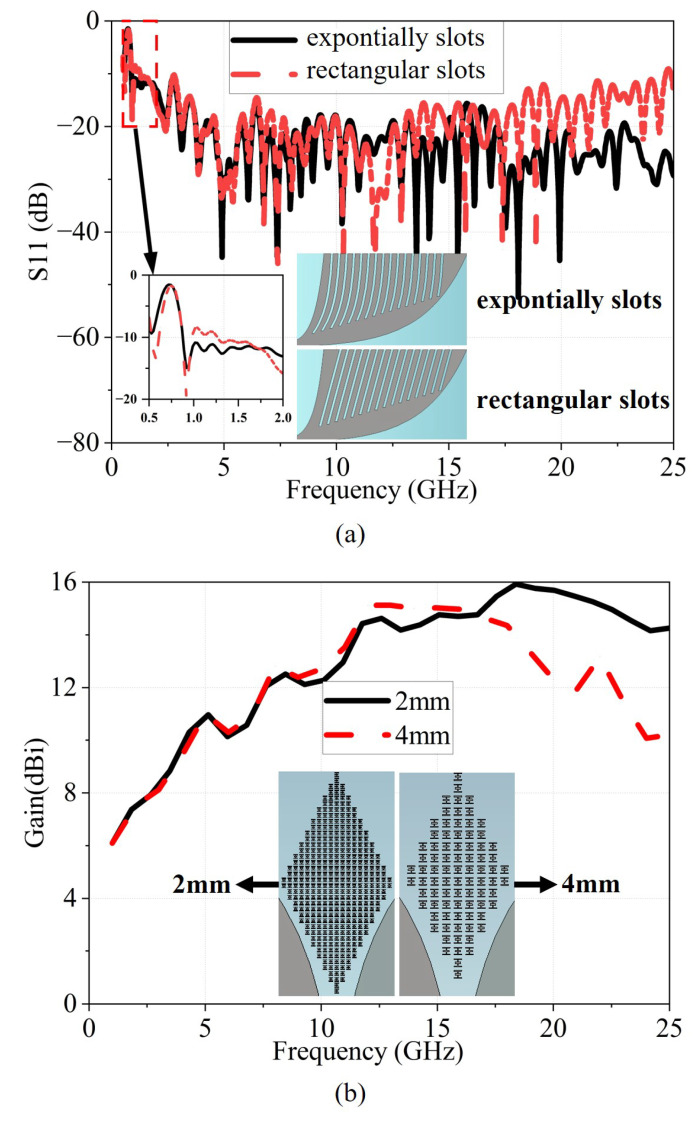
(**a**) Simulated $S_{11}$ parameters of antennas with different slot shapes; the inset shows the two slot geometries used for comparison, (**b**) Simulated gain of antennas with metamaterial units of different sizes; the inset shows the two metamaterial unit-cell array configurations used for comparison.

Figure 5 presents the $S_{11}$ of antennas with various slot shapes, as well as the gain performance when different sizes of metamaterial units are applied. As shown in Figure 5a, when rectangular slots are loaded, the antenna exhibits poor impedance matching at 1.25 GHz with $S_{11}>$ –10 dB. In contrast, the incorporation of exponential slots significantly extends the low-frequency operating bandwidth and achieves better impedance matching in the mid-to-high frequency range. As shown in Figure 5b, the 2 mm and 4 mm metamaterial unit arrays exhibit comparable gain enhancement at low and mid frequencies, both maintaining a high-gain level. However, at frequencies above 17 GHz, the gain enhancement provided by the 4 mm unit array drops sharply, making it difficult for the antenna to maintain a high gain level. In contrast, the 2 mm unit array continues to deliver significant gain improvement in the frequency range above 17 GHz, with the antenna consistently achieving gain levels exceeding 14 dBi.

### Experimental Verification and Comparisons

**Figure 6:**
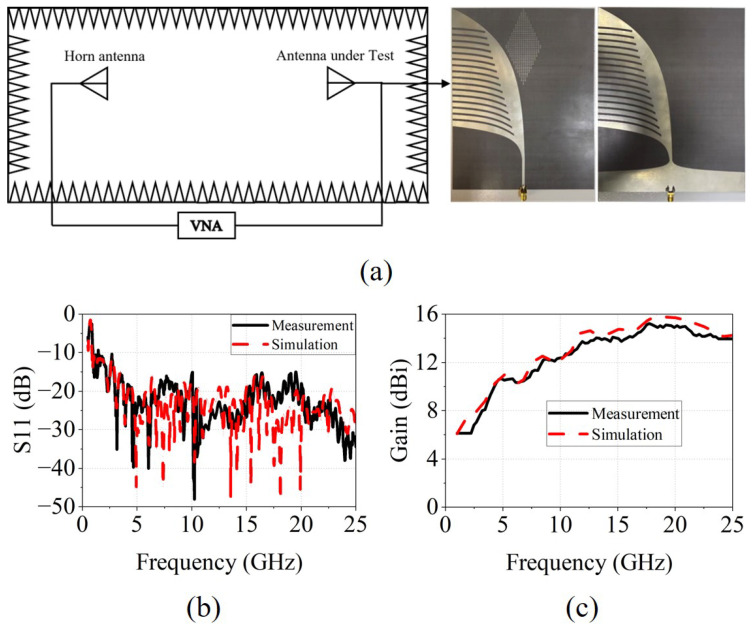
(**a**) Schematic diagram of the experiment setup and the testing sample, (**b**) Measured $S_{11}$ of the antenna, (**c**) Measured gain of the antenna.

A prototype of the proposed antipodal Vivaldi antenna is fabricated and measured. The photograph of the antenna is shown in Figure 6a. The measurement is conducted in a microwave anechoic chamber to ensure an environment free from external electromagnetic interference and unwanted reflections. A vector network analyzer (VNA) is used to record the S-parameters, while a linearly polarized horn antenna serves as the receiving antenna during the far-field gain and radiation-pattern measurements. The proposed antenna is mounted on a low-scattering rotary positioner, allowing its radiation characteristics to be captured over the full angular range. As shown in Figure 6b, the antenna exhibits excellent impedance matching across 1–25 GHz, with $S_{11}$ remaining below –10 dB throughout the band and further suppressed to below –15 dB from 2.2 to 25 GHz. The measured gain, illustrated in Figure 6c, remains above 12 dBi beyond 8.5 GHz, exceeds 14 dBi above 13 GHz, and reaches a maximum of 15.21 dBi at 17.7 GHz.

**Figure 7:**
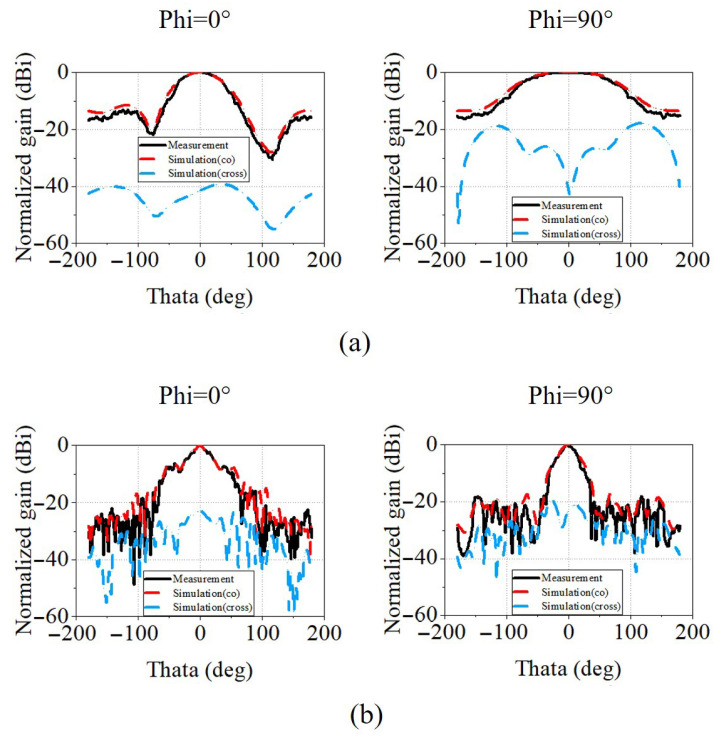

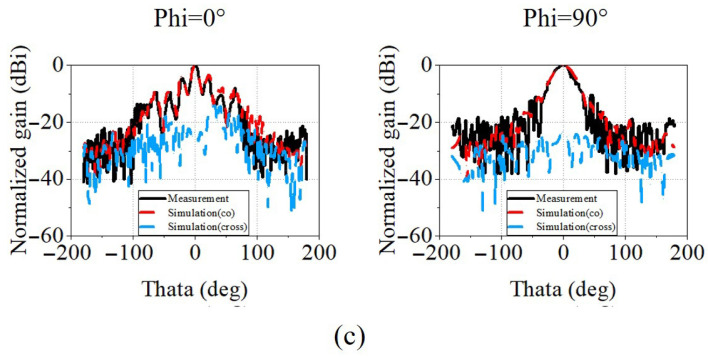
Measured radiation pattern of the antenna at (**a**) 1, (**b**) 13, and (**c**) 25 GHz.

Figure 7a–c present the antenna radiation patterns at 1 GHz, 13 GHz, and 25 GHz, respectively. At all three frequencies, the antenna exhibits stable and symmetric radiation characteristics in both the E-plane and H-plane. Apart from minor fabrication and measurement tolerances, the measured $S_{11}$, gain, and radiation patterns are in good agreement with the simulated results.

In addition, quantitative radiation metrics are extracted from both simulation and measurement. As the frequency increases, the measured half-power beamwidth (HPBW) of the main beam shows a decreasing trend, being approximately 70° at 1 GHz, about 35° at 13 GHz, and further narrowing to around 10° at 25 GHz. The corresponding simulated HPBW values are 71°, 35° and 11°. The measured front-to-back (F/B) ratio is −15.8 dB at 1 GHz, −32.36 dB at 13 GHz, and −31.1 dB at 25 GHz, while the corresponding simulated values are −13.6 dB, −28.1 dB, and −28.8 dB. The first sidelobe level (SLL) also remains reasonably low: the measured SLL is −13 dB at 1 GHz, −16 dB at 13 GHz, and −8 dB at 25 GHz, and the corresponding simulated SLL values are −11 dB, −14 dB, and −8 dB. Across the operating band, the simulated cross-polarization level in the main-beam region remains well suppressed, presenting below −41.5 dB at 1 GHz and below −23 dB at 13 and 25 GHz, confirming good polarization purity. Overall, the simulated and measured results show good agreement, confirming stable endfire directivity with effective suppression of backward radiation and sidelobes across the operating band.

As shown in Table 2, the proposed antenna exhibits a superior balance of ultra-wideband performance, high gain, and compact size when compared with previously reported designs. Specifically, it achieves an impressively wide operating bandwidth of 1 to 25 GHz, covering a broader frequency range than most references, including [11,16,21], and matching or exceeding the performance of typical ultrawideband antennas such as those in [20,22,23]. In terms of gain, the proposed antenna maintains a consistently high level across the entire band, with peak values reaching 15.84 dBi at 18 GHz and remaining above 14 dBi up to 25 GHz, which is notably higher than the gains reported in several prior works, particularly in the high-frequency region. Moreover, these improvements are achieved with a relatively compact structure of 0.5λ×0.6λ, which is smaller than many existing designs such as [20,21,22] that rely on larger apertures. Overall, this work demonstrates that through careful structural optimization, it is possible to simultaneously realize wide bandwidth, high gain, and size reduction, making the proposed antenna highly suitable for integration into modern broadband and multiband communication systems.

## 3. Conclusions

A high-gain, miniaturized antipodal Vivaldi antenna has been proposed in the paper. By introducing tapered exponential slots and a metamaterial array, the design effectively improves impedance matching and enhances gain. The antenna achieves an ultra-wide operating bandwidth from 1 to 25 GHz, a gain of 6.12–15.84 dBi, and a compact size of 0.5λ×0.6λ. Such a combination of ultrawideband matching, high realized gain, and compact size makes the proposed antenna a strong candidate for practical UWB systems, including high-resolution UWB radar and imaging, broadband sensing and spectrum-monitoring platforms, and high-frequency short-range communication front-ends. In these applications, the stable high-frequency gain and improved radiation characteristics enabled by the metamaterial loading help mitigate the gain roll-off and pattern distortion commonly observed in compact Vivaldi antennas, thereby supporting more reliable detection and data transmission in space-constrained environments.

## Figures and Tables

**Table 1:** Geometrical parameters of the proposed antipodal Vivaldi antenna.

Parameter	Value (mm)	Parameter	Value (mm)
*W*	150	$W_{2}$	2
*L*	180	*a*	2
$L_{2}$	19.32	$L_{a}$	1.4
$L_{3}$	35	$W_{a}$	0.14
$L_{4}$	65	$W_{b}$	1.42
$W_{1}$	4	$W_{c}$	0.28

**Table 2:** Comparison of various antennas.

Ref	Operating Band (GHz)	Gain@Freq	Size
[11]	1.3–20	−1.2 dBi @ 1.3 GHz 8.5 dBi @ 10 GHz 9.0 dBi @ 20 GHz	0.27λ × 0.22λ *
[16]	3.2–16.8	4.2 dBi @ 3.2 GHz 10.4 dBi @ 10.5 GHz 6.3 dBi @ 16 GHz	0.35λ×0.35λ
[20]	1–28	5.3 dBi @ 1 GHz 11.6 dBi @ 8 GHz 14.4 dBi @ 18 GHz 11.8 dBi @ 28 GHz	0.6λ×1.3λ
[22]	1–28	2.1 dBi @ 1 GHz 6.0 dBi @ 8 GHz 11.3 dBi @ 18 GHz 8.8 dBi @ 28 GHz	1λ×2λ
[23]	1–10	5.8 dBi @ 1 GHz 15.3 dBi @ 5 GHz 12.9 dBi @ 10 GHz	/
[21]	6–18	10.7 dBi @ 6 GHz 12.7 dBi @ 12 GHz 10.5 dBi @ 18 GHz	1.04λ×1.36λ
This work	1–25	6.09 dBi @ 1 GHz 12.23 dBi @ 10 GHz 15.79 dBi @ 19 GHz 14.26 dBi @ 25 GHz	0.5λ×0.6λ

* *λ* represents the wavelength of the lowest operating frequency.

## Data Availability

Data will be made available on request.
